# MYB24 in control: Transcriptional activation of lignin and cellulose biosynthesis in pear fruit stone cells

**DOI:** 10.1093/plphys/kiad225

**Published:** 2023-04-15

**Authors:** Dyoni M Oliveira

**Affiliations:** Assistant Features Editor, Plant Physiology, American Society of Plant Biologists; Department of Plant Biotechnology and Bioinformatics, Ghent University, 9052 Ghent, Belgium; VIB Center for Plant Systems Biology, 9052 Ghent, Belgium

Pear fruits are economically important worldwide. Stone cells in pear fruits have thickened and lignified cell walls that cause rough flesh texture, reducing fruit quality and economic value (Zhang et al. 2021). Stone cell is a type of sclerenchyma cell in which a secondary cell wall is deposited on the primary wall once the cell expansion is finished. Therefore, the reduction of stone cell content can improve fruit palatability and economic value (Gong et al. 2023).

Lignin and cellulose are the main components of the secondary walls, contributing to the wall stiffness. As a product of the phenylpropanoid pathway, lignin provides mechanical strength and hydrophobicity to the cell (Vanholme et al. 2010; Oliveira et al. 2020). Unlike lignin, cellulose is synthesized by cellulose synthase complexes attached to the plasma membrane, which form cellulose microfibrils in the wall. The biosynthesis of lignin and cellulose is tightly transcriptionally regulated by a large regulatory network in which myeloblastosis (MYB) transcription factors (TFs) directly bind with the promoters of the biosynthetic genes, thereby regulating their expression levels (Zhao and Dixon 2011; Miyamoto et al. 2020). However, the transcriptional network controlling lignin and cellulose biosynthesis in pear fruits remains largely unexplored.

In this issue of *Plant Physiology*, Xue et al. (2023) expand on their previous findings (Wang et al. 2021) to identify a new *MYB* gene, *PbrMYB24*, and demonstrate its functions as a transcriptional activator of the biosynthesis of lignin and cellulose in stone cells of pear fruit (*Pyrus bretschneideri*). In their earlier study, the authors constructed a co-expression network of genes involved in secondary cell wall formation based on gene expression profiles of 206 pear cultivars and identified 8 hub genes encoding MYB TFs putatively involved in regulating secondary cell wall biosynthesis (Wang et al. 2021). Here, Xue et al. combined the analysis of gene expression profiles and transcriptomics of several pear cultivars with varied stone cell contents with functional studies to dissect the function of PbrMYB24 in stone cells.

First, RNA-seq datasets of pear fruits were used to demonstrate that *PbrMYB24* expression levels were correlated with stone cell contents. Based on the co-expression network analysis, they observed that *PbrMYB24* was clustered with lignin and cellulose biosynthetic genes as well as 2 TFs, *PbrMYB169* and *PbrNSC*. Next, the phylogenetic analysis showed that PbrMYB24 is related to other MYB TFs predicted to regulate lignin biosynthesis across different species, and it clusters with AtMYB46, a known activator of secondary cell wall biosynthesis in Arabidopsis (Zhong et al. 2007), suggesting that PbrMYB24 might have an analogous function in pear fruit stone cells.

To test the function of PbrMYB24 in the biosynthesis of lignin and cellulose in pear fruit stone cells, the authors used a broad range of genetic and biochemical approaches. They transiently overexpressed and silenced *PbrMYB24* in pear fruit flesh and determined the cellulose and lignin deposition in the stone cell tissues. The transient overexpression of *PbrMYB24* induced higher transcript levels of genes involved in lignin and cellulose biosynthesis and consequently more incorporation of these polymers in the wall. Silencing *PbrMYB24* induced exactly the opposite effects (Xue et al. 2023).

Consistent with the observation that PbrMYB24 can simultaneously regulate lignin and cellulose deposition in pear fruit stone cells, Xue et al. introduced *PbrMYB24* into Arabidopsis to unequivocally confirm its role in lignin and cellulose biosynthesis. Heterologous expression of *PbrMYB24* in Arabidopsis led to increased transcript abundance of genes involved in lignin and cellulose biosynthesis, with consequent higher lignification and cellulose deposition in transgenic stems. Transmission electron microscopy analysis of transgenic Arabidopsis stems evidenced that the secondary cell walls of interfascicular fiber and vessels were thicker than in the wild type. These results demonstrated that *PbrMYB24* not only simultaneously promotes both lignin and cellulose biosynthesis in pear flesh and callus tissue but also in *PbrMYB24-*expressing Arabidopsis stems.

Synthetic plant hormones, including the auxin-mimicking 2,4-D and the synthetic cytokinin 6-benzyl-aminopurine, are typically used to induce plant growth and development of transformed callus, although they can also affect the biosynthesis of secondary cell wall components (Pesquet et al. 2019). Here, pear calli overexpressing *PbrMYB24* and growing in a medium containing 2,4-D and 6-benzyl-aminopurine exhibited a nearly complete depletion of lignification. To overcome this challenge, the authors successfully established a new lignin induction system for pear calli by supplementing the medium with epibrassinolide, an inductor for the differentiation of suspension cells to tracheary elements (Pesquet et al. 2019). Notably, the *PbrMYB24*-overexpressing pear callus displayed the lignin-derived red color by phloroglucinol-HCl staining, indicating that lignification occurred in the callus as well.

Previous findings have shown that MYB proteins bind to *cis*-elements, including AC elements and MYB-binding sites, to regulate secondary cell wall formation (Zhao and Dixon 2011; Geng et al. 2019). To specifically determine the regulatory relationship between PbrMYB24 and genes involved in secondary cell wall biosynthesis, the authors used a dual-luciferase reporter assay to clearly show that PbrMYB24 activates the promoters of genes involved in the phenylpropanoid pathway, lignin polymerization, and cellulose synthase genes *CESA8a* and *CESA8b.* Next, the authors used yeast one-hybrid assays to demonstrate that PbrMYB24 specifically binds to AC elements in the promoters of lignin biosynthesis genes and MYB-binding sites of cellulose synthase genes to promote secondary cell wall biosynthesis (Figure).

**Figure.**
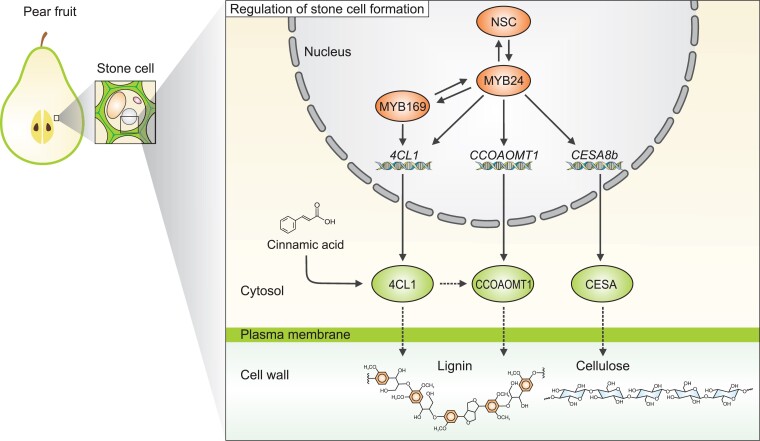
A model of MYB24 activation of secondary cell wall formation in pear fruit stone cells. PbrMYB24 protein binds to the promoters of *MYB169* and *NSC*, and both MYB169 and NSC proteins can activate *MYB24* expression. MYB24 directly activates the transcription of *4CL1*, *CCOAMT1*, and *CESA8b* genes activating the biosynthesis of lignin and cellulose.

Because the co-expression network revealed that *PbrMYB24* is clustered with *PbrMYB169* and *PbrNSC*, the authors investigated the mutual interactions and activations among the 3 TFs. They combined dual-luciferase reporter assays and firefly luciferase complementation imaging in which tobacco leaves were co-infiltrated with MYB24-cLUC, MYB169-nLUC, or NSC-nLUC constructs. The results showed that PbrMYB24 protein did not bind with PbrMYB169 and PbrNSC. They showed that PbrMYB24 can activate the promoters of *PbrMYB169* and *PbrNSC* genes, and *PbrMYB24* promoter can be activated by PbrMYB169 and PbrNSC. Interestingly though, self-activation of *PbrMYB24* was not observed. Based on these results, the authors suggested that PbrMYB24, PbrMYB169, and PbrNSC function in a coordinated manner to regulate lignin and cellulose biosynthesis in pear fruit stone cells (Figure).

In conclusion, the work of Xue et al. (2023) provides insights into the function of *PbrMYB24* as a transcriptional activator of lignin and cellulose biosynthesis in pear fruit stone cells. With these findings, further studies could also use *PbrMYB24* as a bait gene to retrieve interacting TFs to construct a more detailed transcriptional regulatory network of secondary cell wall biosynthesis in stone cells. In addition, the authors’ findings not only show that the plant hormone induction system is useful for observing the phenotype in pear calli but also provide a basis for exploring the function and application of plant growth regulators in the regulation of stone cell formation.
